# Characteristics of Fatal, Pedestrian-Involved, Motor Vehicle Crashes in West Virginia: A Cross-Sectional and Spatial Analysis

**DOI:** 10.3390/ijerph20075251

**Published:** 2023-03-24

**Authors:** Toni M. Rudisill, Lauren Olivia Barbee, Brian Hendricks

**Affiliations:** 1 Department of Epidemiology and Biostatistics, West Virginia University, Morgantown, WV 26506, USA; 2 Department of Forensic and Investigative Science, West Virginia University, Morgantown, WV 26506, USA

**Keywords:** pedestrian, fatal, motor vehicle, rural

## Abstract

Fatal, pedestrian-involved, motor vehicle collisions are increasing in the United States yet remain lower in rural states such as West Virginia. This study’s purpose was to investigate the overall risk factors of pedestrian fatalities by rurality and sex in West Virginia. Data were obtained from the Fatality Analysis Reporting System. The fatality had to occur within West Virginia between 1 January 2009 and 31 December 2019. Risk factors of rural vs. urban and male vs. female crashes were determined using multivariable logistic regression models. Clustering of crash locations was analyzed using kernel density estimation and Ripley’s K. Among the 254 fatalities, most victims were male (70%). Most crashes occurred at night (76%), on highways (73%), on level (71%), non-curved (84%), dry (82%) roads during fair weather conditions (82%). Nearly 34% of the victims tested positive for alcohol. Men were 2.5 times as likely to be hit in a rural area (OR = 2.5; 95% CI 1.2, 5.4), on curved roads, and 57% less likely (OR = 0.43; 95% CI 0.2, 0.9) to test positive for drugs compared to women. Crash characteristics, including location, were similar between the sexes. As many risk factors were modifiable behaviors, public health interventions to ensure pedestrian safety may be necessary.

## 1. Introduction

Between 2010 and 2019, ~724,000 pedestrians were injured and 53,494 were killed in motor vehicle collisions in the United States (US) [1]. During this time period, fatal pedestrian-involved motor vehicle collisions increased 44% nationally while the estimated number of walking trips and vehicle miles traveled remained constant [2,3]. While the exact reasons for this disparity are unknown, findings from previous research suggest that increases in population density, urban sprawl, increased homelessness, greater employment density, increases in the horsepower and/or size of vehicles, greater per capita alcohol consumption, and driver or pedestrian distraction due to cellphones may have contributed to the increase in pedestrian fatalities [4,5,6,7,8,9,10,11,12,13,14,15].

While pedestrian fatality rates typically trend higher in states with warmer climates and/or seasonal tourism, such as Florida, California, Hawaii, and Arizona, pedestrian fatality rates tend to run lower in rural, less populated states, such as West Virginia [1,16]. For example, a study conducted in West Virginia using data from 2008–2012 found that the overall traffic fatality rate was 71% higher than that of the rest of the US, but pedestrian fatality rates were 26% lower than the national rate; the authors’ attributed this finding to the state’s mountainous topography and environment, such as steep inclines, narrow roads, poor lighting, and lack of cellphone reception in case of emergencies, which may dissuade individuals from walking [16]. Previous studies revealed that the built environment, even in rural areas, can greatly impact how much individuals walk [4,17]. Areas with mixed land use, destinations to walk to, and perceived neighborhood and traffic safety (i.e., quietness, appealing esthetics, low crime, low traffic volumes, presence of maintained sidewalks, intersections, adequate lighting, etc.) can influence travel behavior [4,17]. Specifically, if individuals feel unsafe or do not find their environment appealing, they are less likely to walk.

However, in rural states, such as West Virginia, many individuals may still need to walk as a primary means of transportation regardless of their environmental perception. Research shows that ~11% of impoverished, rural households in the US do not have access to a personal vehicle [18]. The highest percentages of “vehicle-less” homes are found in the rural South, Southwest, Alaska, and Appalachia—which includes West Virginia [19]. To complicate matters, many rural areas also lack public transportation [20]. Without access to a personal vehicle or public transportation, many individuals in rural areas must walk to complete activities of daily living, which increases their risk of being involved in a motor vehicle collision.

To date, very few studies have investigated risk factors of rural, pedestrian-involved motor vehicle crashes [21,22,23,24,25,26]. Separate studies conducted in Illinois, Nebraska, Alabama, Connecticut, and North Carolina collectively found that low lighting, lack of traffic control devices, roadway classification, road width or the number of lanes, weather, pedestrian/driver alcohol consumption, higher speeds, larger vehicles, commercialized areas, non-intersections, and roads with lack of shoulders were all associated with rural collisions involving pedestrians [21,22,23,24,25,26].

Despite the risk factors identified in previous studies, virtually no studies have investigated the differences in fatal, pedestrian-involved, motor vehicle collisions in general or by sex in West Virginia. Thus, the purpose of this study was to (1) describe and determine risk factors that predicted fatal pedestrian crashes overall and in rural vs. urban areas of West Virginia, (2) compare male vs. female pedestrian crashes, and (3) discern where these collisions were occurring through a cross-sectional and spatial analysis. Because men and women have different travel behaviors and risk tolerances, it was believed that crash characteristics and locations would differ between the sexes [27,28,29,30,31]. It was hypothesized that crashes involving female pedestrians would occur more frequently during the daytime, on weekdays, on primary roads near towns, in favorable weather conditions compared to males due to the fact that females are typically more safety conscious [27]. These findings could greatly inform future transportation safety initiatives in West Virginia and potentially other rural areas.

## 2. Materials and Methods

### 2.1. Study Design and Data Source

This was a cross-sectional study. The data for this analysis were obtained from the National Highway Traffic Administration’s Fatality Analysis Reporting System (FARS). The FARS is publicly available and encompasses data taken from all fatal crashes that occur on public roadways in all 50 states and U.S. territories. To be included in the dataset, at least one individual involved in a crash has to die within 30 days of the incident. The FARS dataset has been described in more detail elsewhere [32]. 

### 2.2. Study Population

The study population was limited to any pedestrian who was fatally injured in a motor vehicle collision, which occurred within the state of West Virginia, from 1 January 2009 to 31 December 2019. 

### 2.3. Data Management

The two primary dependent variables in this study were the rurality of the crash (i.e., yes/no), which was based on the functional classification of the roadway where the crash occurred, and the reported sex of the pedestrian. Numerous independent variables were created for this analysis and are shown in Table 1. These variables were chosen because they were previously investigated in studies concerning pedestrian injuries or fatalities [21,22,23,24,25,26,33]. The variable survival time was calculated by subtracting the pedestrians’ time of death from the approximate time that their crash occurred (in minutes). A pedestrian was considered positive for alcohol or drugs (i.e., yes/no) if any alcohol (i.e., ≥0.01 mg/mL) or drugs were detected via an administered urine and/or blood test. Drugs included prescription, non-prescription, and illicit substances. Drug test results did not include nicotine, aspirin, or drugs administered to the pedestrian post-collision. If the pedestrian was hit by a driver who held a West Virginia driver’s license, they were considered to be hit by an in-state driver (i.e., yes/no). If the driver who struck the pedestrian was involved in a previous collision in the previous 5 years, or received a license suspension, driving-while-intoxicated citation, or speeding or other traffic violation in the previous 3 years, then they were considered to be previously cited for a traffic infraction (i.e., yes/no). If the driver was not present at the scene of the crash as per the collision report, then the crash was considered a ‘hit and run’ (i.e., yes/no). The variable ‘light conditions’ described the lighting at the time of the crash. If the crash occurred during daytime, night, or during the night on a street with no lights, it was considered a light or dark crash, respectively. The ‘low light’ classification was combined those who experienced a collision at dawn, dusk, or at night under a lighted street due to small sample sizes. Weather conditions were considered inclement if any precipitation or excessive wind was occurring at time of the collision (i.e., yes/no). Road surface conditions described precipitation on the roadway at time of collision; this was classified as dry vs. wet, frozen, or other due to sample size. Wet surfaces included wet but not frozen roadways or the presence of moving or standing water. Frozen surfaces included roadways that had snow, slush, ice, or frost on them. Roadways that contained sand, dirt, mud, oil, or other conditions were all categorized as ‘Other’. Highway crashes (i.e., yes/no) were defined as crashes which occurred on interstates, U.S. routes, or state routes. Crashes which occurred at intersections (i.e., yes/no) were determined using the location of the pedestrian relative to the road when the crash transpired. Road curvature (i.e., yes/no) was determined based on the alignment of the roadway where the crash occurred; curved roads could arc left or right. The slope of the roadway was classified as level vs. grade (i.e., sloped), top of hill (i.e., hill crest), or sag (i.e., bottom of hill), which was combined due to small sample sizes and based on the road profile where the crash arose. The latitude and longitude of the crashes were used for the spatial analyses.

### 2.4. Statistical Analyses

Descriptive statistics (e.g., means, standard deviations, frequencies, percentages) were used to compare population characteristics. Characteristics were stratified separately by sex and by rurality. Due to sheer sizes of the tables, descriptive statistics of rural vs. urban crashes are included in Appendix A.

Given that the dependent variables were dichotomous and the study’s purpose was to determine what characteristics predicted the odds (and resulting 95% confidence intervals) of rural vs. urban crashes and then male vs. female crashes, multivariable logistic regression was utilized; logistic regression is similar to linear regression except that it utilizes a logit link to model the probability of an event occurring vs. not occurring [34]. The resulting odds ratios produced from these models are simply one group’s odds of experiencing the desired outcome compared to the referent group’s odds.

Prior to running the regression analyses, all independent variables noted, including year of the crash as a continuous variable, were assessed for collinearity using the variance inflation factor and tolerance. No collinearity was observed between independent variables. Year of crash was included in the models as it could be a confounder of the relationship between the covariates and dependent variables given the multiple years of data analyzed. All independent variables and crash year were then entered into the multivariable logistic regression model using backward selection with the *p*-value ≤ 0.15; this *p*-value was chosen as it has been shown to be the most efficient for models using backward selection [35]. Hosmer Lemeshow Goodness-of-Fit tests were used to assess model fit. McFadden’s R-square was calculated and presented in the regression tables; this statistic is a pseudo-R square, which is similar to a R-square in linear regression and estimates the variance explained by the model, except that it is specifically for logistic regression. Because >95% of crashes involved a single pedestrian, correlation between crashes was not accounted for. Hypothesis tests were two-sided with α = 0.05. These analyses were conducted using SAS software version 9.4.

For the spatial analyses, geographic patterns in the occurrence of fatal male and female pedestrian injuries were analyzed separately using isotropic kernel density estimation (KDE) leveraging each events’ latitude and longitude with a WGS84 projection. KDE analyses were conducted in the spatstat package in R [36], and included the Jones Diggle improved edge correction, which has been shown to reduce distortion of density estimates nearer to the edge of boundaries (e.g., West Virginia’s state border), and a fixed bandwidth [37,38]. Previous studies have demonstrated that fixed bandwidths are ideal when estimating event density but have a limited ability to infer risk for sparsely populated areas [39,40,41]. In this study, we were primarily concerned with understanding patterns in the number of events; therefore, fixed bandwidth appropriately captured spatial trend in fatal pedestrian crashes [39]. The difference in Ripley’s K function was assessed to describe differences in clustering of fatal pedestrian injuries across West Virginia by sex. Briefly, Ripley’s K is a method for describing whether points are dispersed, random, or clustered. The difference in K functions provides an opportunity to understand whether there is excess in clustering of events (e.g., fatal pedestrian injuries among males) given the distribution of a comparison group (e.g., fatal pedestrian injuries among females) [42]. The difference in K functions was assessed using the smacpod package in R and results were permuted 999 times using Monte Carlo simulations [43]. Statistical significance in clustering of fatal pedestrian injuries among males compared to females was assessed at the 0.05 alpha level. 

## 3. Results

From 2009 to 2019, 254 pedestrians were fatally injured in motor vehicle collisions in West Virginia (Table 1). Nearly 71% (N = 179) were male. The mean survival time for women post-collision was much lower than that for males (i.e., 685 vs. 1232 min, respectively). Female victims tended to be younger than males and nearly 95% of victims were of white race. Over one-third of victims tested positive for alcohol and more females tested positive for drugs compared to males (i.e., 55% vs. 36%, respectively). The majority of crashes occurred Monday–Friday (69%), in the evenings (76%), on highways (73%), on level (71%), non-curved (84%), dry (82%) roads during fair weather conditions (82%). Descriptive statistics stratified by rurality are shown in Appendix A Table A1.

The results of the multivariable logistic regression model comparing the adjusted odds of experiencing a rural fatal pedestrian crash to the odds of an urban fatal pedestrian crash are shown in Table 2. Only pedestrian sex and whether the crash occurred on a highway significantly predicted a rural crash. After adjusting for covariates, rural crashes were 2.6 times as likely to involve a male pedestrian (OR = 2.6; 95% CI 1.2, 5.5). Likewise, after adjusting for covariates, rural crashes were 66% less likely to occur on a highway (OR = 0.34; 95% CI 0.14, 0.82).

The results of the multivariable logistic regression model comparing the adjusted odds of a male fatal pedestrian crash compared to the odds of a female fatal pedestrian crash are shown in Table 3. The analyses determined that men were 2.5 times as likely to be hit in a rural area (OR = 2.5; 95% CI 1.2, 5.3), on curved roads, and 56% less likely (OR = 0.44; 95% CI 0.22, 0.89) to test positive for drugs compared to women (Table 3).

**Figure 1:**
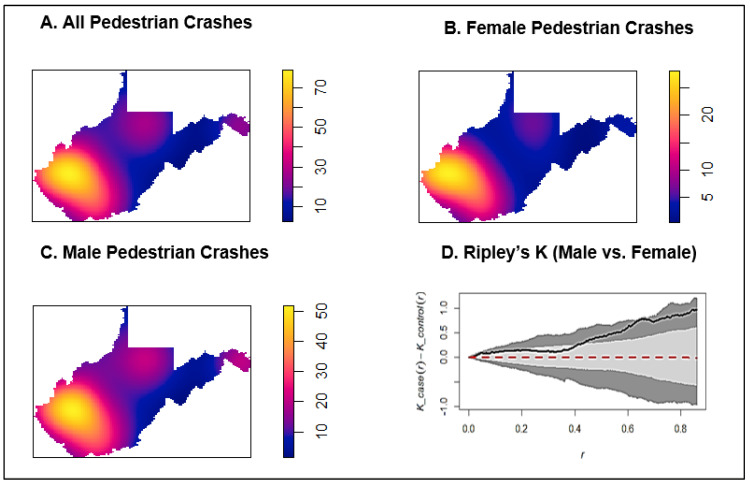
Kernel density estimations (KDEs) of all pedestrians, male, and female crashes along with Ripley’s K comparing intensity of clustering between male vs. female crashes in West Virginia, 2009–2019. Upper-left (**A**): KDE of all pedestrian crashes. Upper-right (**B**): KDE of female pedestrian crashes. Lower-left (**C**): KDE of male pedestrian crashes. Lower-right (**D**): Ripley’s K comparing intensity of clustering between male and female crashes. For the KDE (panels (**A**–**C**)), areas of purple have lower numbers of fatal crash sites, and bright yellow have a higher number of crash sites.

**Figure A1:**
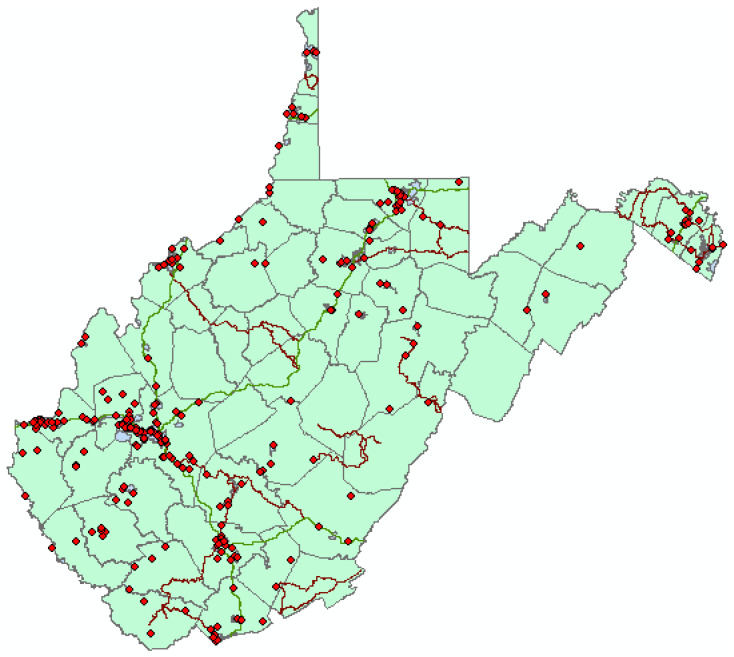
Map of all pedestrian fatalities in WV 2009–2019. Green and burgundy lines represent major roadways. Red diamonds represent individual crashes. Blue-grey areas represent designated towns or cities.

**Figure A2:**
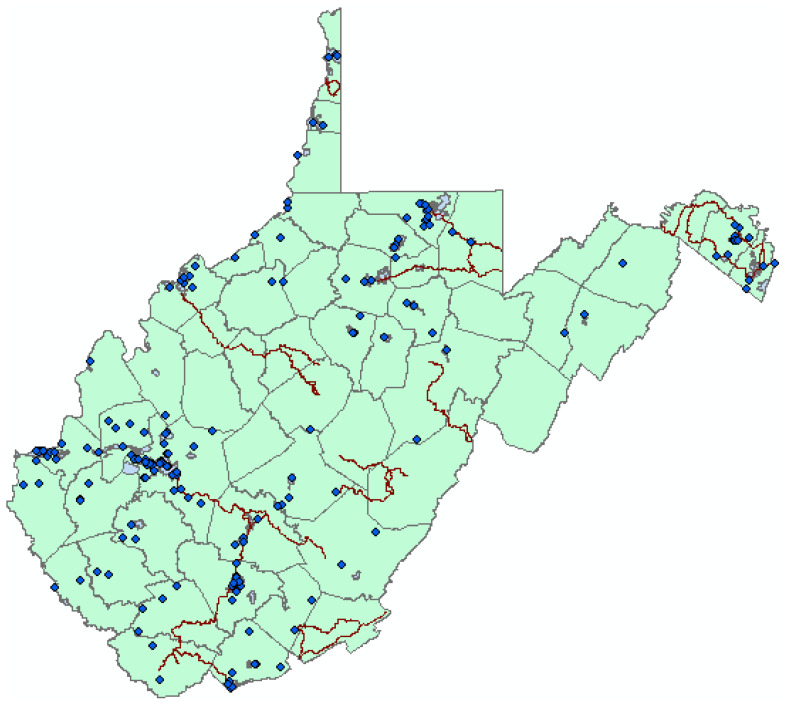
Map of male pedestrian fatalities in WV, 2009–2019. Burgundy lines represent major roadways. Blue diamonds represent individual crashes. Blue-grey areas represent designated towns or cities.

**Figure A3:**
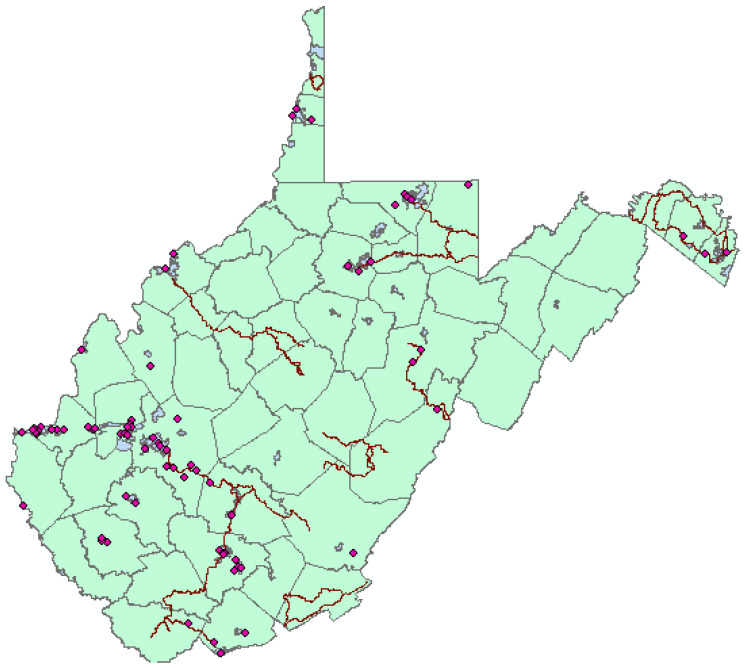
Map of female pedestrian fatalities in WV, 2009–2019. Burgundy lines represent major roadways. Pink diamonds represent individual crashes. Blue-grey areas represent designated towns or cities.

Results of the spatial analyses showed that crash locations were similar for males and females; upon visual inspection, most crashes occurred along major roadways directly outside of towns (Figure A1, Figure A2 and Figure A3). Kernel density estimations of both sexes and overall showed that the majority of pedestrian fatalities were clustered in southern West Virginia (Figure 1). Males also tended to experience more collisions in the North-Central and the Eastern panhandle of the state. The intensity of crashes was also significantly higher in males relative to the spatial pattern of females (panel D, Figure 1).

## 4. Discussion

While West Virginia’s topography makes it unique from many other states (i.e., the entire state lies with the Appalachian mountain range), this study found that several risk factors associated with fatal, pedestrian-involved, motor vehicle crashes were comparable to those seen in previous studies conducted in other states. While crash characteristics were generally similar for urban and rural crashes, there were slight differences observed between male and female pedestrians. From a public health perspective, numerous opportunities exist to make pedestrian travel safer in the Mountain state.

This study determined that many pedestrian-involved, fatal crashes in West Virginia occurred at night or during low light conditions, on highways, during fair weather conditions, at non-intersections, on straight and level roads directly outside of towns. Moreover, many pedestrians had tested positive for alcohol at the time of the crash. Several other studies conducted in rural areas in other states showed similar findings. For example, the US Federal Highway Administration conducted a study in 2003 which investigated rural, fatal pedestrian crashes in ten states (i.e., Arizona, California, Colorado, Florida, Louisiana, Montana, New Mexico, Oregon, Texas, and Wyoming) [44]. That study’s findings were virtually identical to those of the present study. Thus, despite the mountainous terrain, which often discourages many from walking, the findings in West Virginia were similar to those in other states. In terms of crash location, it is logical that many of the fatalities occurred in relative proximity to towns and may be reflective of population density. Many West Virginia towns, albeit small in size, offer places for pedestrians to walk to such as post offices, general stores, and bars/restaurants. The clustering of fatal crashes in the southern part of the state may be due to increased distance from the site of the collisions to healthcare facilities and/or longer emergency medical service response times in this area; while the exact reason is unknown, it is worthy of additional research.

The differences observed between male and female pedestrians in this study are also supported by the extant literature. Specifically, this study found that the majority of pedestrians were male (i.e., 70%) and that males were more likely to be struck on rural or curved roads than females. Previous studies have also shown that males are over-represented in pedestrian fatalities; a study conducted among 10 states by the Federal Highway Administration also found that 69% of pedestrian fatalities were male [44]. This disparity could be due to differences in travel behaviors and risk tolerances between the sexes. Previous studies have shown that women often walk more than men, especially when commuting to work [45]. This implies that women may spend more time being at risk of pedestrian–motor vehicle encounter, yet more male pedestrians are killed. This disparity could be driven by the fact that women take less risks and may practice safer traffic behaviors [46]. For example, a previous study examined the road crossing behavior of both males (N = 500) and females (N = 500) at intersections during a high-risk situation where a vehicle was approaching; the study found that men were three times as likely to cross the street during this high-risk situation compared to females [47]. This fact may explain why women were less likely than males to be hit on rural or curved roads; females may have perceived these conditions as riskier and chose not to walk there.

Despite the slight differences seen between males and females concerning rurality and road curvature, their crash characteristics were generally similar. It was hypothesized that because females typically are more safety conscious than males, that a majority of female pedestrian fatalities would occur during ‘safer’ days, times, locations, and weather conditions. This was not entirely the case. Almost 80% of the fatalities occurred during hours where lighting and visibility could have been poor for drivers. It is possible that the women who were hit and killed may have been fundamentally different from the average female pedestrian, but this is unknown. Moreover, the female pedestrians in this study were significantly more likely to be drug positive than males. Due to the limitations of the FARS data, it should be noted that the types or specific drugs were not investigated [48]. Previous studies have shown that females typically consume more prescription medication than males; a study conducted in 2010 showed that women were significantly more likely to use one or more daily prescription medications compared to men [49]. It is possible that some of these drugs were prescriptions, but this is unknown.

From a public health perspective, ample opportunities for intervention exist to make pedestrian travel safer in West Virginia. First, many of the pedestrians in this study were killed at night. If pedestrians must walk at night due to transportation issues, they can protect themselves by being more visible to drivers. This could include wearing bright-colored clothes, reflective material, or carrying flashlights and walking against oncoming traffic. Secondly, many of the drivers who hit the pedestrians had recent traffic citations, including for speeding. Drivers must slow down within and directly outside of towns/cities as this is where people are more apt to be walking. Lowering speed limits around towns/cities and active enforcement of speed limits have been shown to reduce pedestrian fatalities [50].

While West Virginia’s mountainous terrain can be challenging from a traffic engineering perspective, numerous opportunities exist to protect pedestrians. The United States Department of Transportation has supported the adoption and implementation of ‘Complete Streets’ policies in communities for well over a decade [51,52]. These urban planning policies encourage safety, equity, and connectivity for all road users [51]. For pedestrians, constructing more sidewalks or adding shoulders to roads entering towns/cities or directly outside of them provides space for pedestrians to walk. Streetlights can be added to the outskirts of town/cities to ensure pedestrians are seen by drivers. Installing signage outside of towns/cities to warn drivers of pedestrians might help; traffic calming measures such as speed bumps, roundabouts, road medians/islands, and travel lanes for pedestrians are all effective countermeasures to reduce pedestrian fatalities [50,53]. Given the number of ‘vehicle-less’ households in West Virginia [19], increased public transportation access around more populated areas could also help keep individuals off unsafe roads. Increased cellphone service could also improve drivers’ ability to contact individuals for roadside assistance in the event of car trouble and keep them from walking rural roads. Many of these changes are encouraged in ‘Complete Streets’ policies [51,52]. However, these changes take time to implement, require public and legislative support, and, more importantly, funding, which is not always available in an impoverished state such as West Virginia, to ensure their adoption, implementation, and evaluation.

### Limitations

While the strengths of this study are that it utilized 11 years of crash data and investigated risk factors of rural, fatal pedestrian crashes overall and by rurality and sex, both cross-sectionally and spatially, it is not without limitation. First, this analysis did not look at who was at fault in the crash (i.e., the pedestrian or the driver). Secondly, females were under-represented in the crashes; thus, the sample size may not have been large enough to detect a significant difference between the sexes. Moreover, due to limitations of the FARS data, the types of drugs that pedestrians consumed were not investigated and the variable was broadly classified [48]. Lastly, the findings of this study may not apply to non-fatal, but injurious pedestrian crashes, nor may be generalizable to other states.

## 5. Conclusions

Despite its mountainous terrain, this study found that numerous risk factors associated with fatal, pedestrian-involved motor vehicle collisions previously identified in other states were also observed in West Virginia. These findings could greatly inform public health interventions within this state. Future research could involve designing and implementing such interventions and evaluating their effectiveness. Likewise, this study also found that numerous opportunities exist at both societal and individual levels to ensure safer pedestrian travel. While public health campaigns, increased public transportation, or traffic engineering projects could protect pedestrians, they often take time and/or funding to implement. In the interim, given that many of the risk factors identified were modifiable behaviors, pedestrians and drivers can take precautions to minimize these tragedies. If pedestrians can increase their visibility while drivers reduce speed and maintain vigilance near towns, fatal pedestrian–vehicle encounters may be reduced.

## Figures and Tables

**Table 1:** Characteristics of fatal pedestrian crashes that occurred in West Virginia, 2009–2019 stratified by sex ^a.^

	Male (N = 179)		Female (N = 75)		Total (N = 254)	
Characteristic	Mean	SD	Mean	SD	Mean	SD
Survival Time (min)	1232	4315	685	2709	1074	3920
	N	%	N	%	N	%
Pedestrian Age (years)						
<20	12	6.70	5	6.67	17	6.69
20–29	28	15.64	16	21.33	44	17.32
30–39	34	18.99	17	22.67	51	20.08
40–49	38	21.23	14	18.67	52	20.47
50–59	31	17.32	8	10.67	39	15.35
≥60	36	20.11	15	20.00	51	20.08
Pedestrian Race						
White	154	95.65	57	91.94	211	94.62
Non-white	7	4.35	5	8.06	12	5.38
Missing	18		13		31	
Pedestrian Positive for Alcohol						
No	110	66.27	42	63.64	152	65.52
Yes	56	33.73	24	36.36	80	34.48
Missing	13		9		22	
Pedestrian Positive for Drugs						
No	106	63.86	30	45.45	136	58.62
Yes	60	36.14	36	54.55	96	41.38
Missing	13		9		22	
Hit by an In-state Driver						
No	24	14.20	14	19.44	38	15.77
Yes	145	85.80	58	80.56	203	84.23
Missing	10		3		13	
Driver Previously Cited for Traffic Infractions						
No	94	56.63	39	56.52	133	56.60
Yes	72	43.37	30	43.48	102	43.40
Missing	13		6		19	
‘Hit and Run’ Crash						
No	147	82.12	61	81.33	208	81.89
Yes	32	17.90	14	18.67	46	18.11
Vehicle that Struck Pedestrian						
Car	55	32.74	20	28.17	75	31.38
Sport Utility Vehicle	38	22.62	16	22.54	54	22.59
Light Truck/Van	51	30.36	25	35.21	76	31.80
Other	24	14.29	10	14.08	34	14.23
Missing	11		4		15	
Season of Crash						
Dec–Feb	34	18.99	18	24.00	52	20.47
Mar–May	35	19.55	15	20.00	50	19.69
Jun–Aug	52	29.05	17	22.67	69	27.17
Sept–Nov	58	32.40	25	33.33	83	32.68
Day of Crash						
Mon–Fri	124	69.27	50	66.67	174	68.50
Sat–Sun	55	30.73	25	33.33	80	31.50
Time of Crash						
6:00 AM to 4:49 PM	44	24.72	16	21.62	60	23.81
5:00 PM to 5:59 AM	134	75.28	58	78.38	192	76.19
Missing	1		1		2	
Light Conditions						
Light	43	24.02	14	18.67	57	22.44
Low light	36	20.11	21	28.00	57	22.44
Dark	100	55.87	40	53.33	140	55.12
Inclement Weather						
No	146	81.56	62	82.67	208	81.89
Yes	33	18.44	13	17.33	46	18.11
Road Surface Conditions						
Dry	146	82.49	60	80.00	206	81.75
Wet/frozen/other	31	17.51	15	20.00	46	18.25
Missing	2		0		2	
Highway Crash						
No	49	27.37	19	25.33	68	26.77
Yes	130	72.63	56	74.67	186	73.23
Rural Crash						
No	91	51.12	51	68.92	142	56.35
Yes	87	48.88	23	31.08	110	43.65
Missing	1		1		2	
Crash Occurred at Intersection						
No	150	88.24	62	87.32	212	87.97
Yes	20	11.76	9	12.68	29	12.03
Missing	9		4		13	
Roadway Speed Limit						
1–35 MPH	49	28.16	22	30.56	71	28.86
36–55 MPH	89	51.15	40	55.56	129	52.44
56 + MPH	36	20.69	10	13.89	46	18.70
Missing	5		3		8	
Roadway Curved						
No	146	81.56	68	90.67	214	84.25
Yes	33	18.44	7	9.33	40	15.75
Slope of Roadway						
Level	123	69.10	56	74.67	179	70.75
Grade/hill/sag	55	30.90	19	25.33	74	29.25
Missing	1		0		1	

Abbreviations: MPH = miles per hour; SD = standard deviation; ^a^: Percentages may not add to 100% due to rounding.

**Table 2:** Factors that predict rural vs. urban fatal, pedestrian, motor vehicle crashes in West Virginia, 2009–2019 ^a.^

Independent Variable	OR	95% CI	*p*
Male Pedestrian			0.015
Yes	2.57	1.20, 5.49	
No	1.00	Referent	
Pedestrian Race			0.075
White	8.91	0.80, 98.92	
Other	1.00	Referent	
Pedestrian Positive for Alcohol			0.147
No	1.00	Referent	
Yes	1.72	0.83, 3.56	
Time of Crash			0.078
6:00 AM to 4:49 PM	1.00	Referent	
5:00 PM to 5:59 AM	0.40	0.15, 1.11	
Light Conditions			0.098
Light	1.00	Referent	
Dawn/Dusk	0.79	0.25, 2.54	
Dark	2.05	0.68, 6.17	
Highway Crash			0.017
No	1.00	Referent	
Yes	0.34	0.14, 0.82	
Roadway Speed Limit			0.124
1–35 MPH	1.00	Referent	
36–55 MPH	2.45	1.02, 5.89	
56 + MPH	2.50	0.83, 7.57	

Abbreviations: CI = confidence interval; MPH = miles per hour; OR = odds ratio; *p* = probability value; ^a^: A multivariable logistic regression model was run using backward selection with the *p*-value = 0.15 for an independent variable to remain in the model. Model initially included pedestrians’ ages, sex, race, alcohol positivity, drug positivity, whether they were struck by an in-state driver, whether driver was previously cited, whether crash was a hit and run, vehicle type, season of crash, whether crash was on a weekday, time of crash, light conditions, weather conditions, road surface conditions, whether crash occurred on highway, whether crash occurred at an intersection, speed limits, whether crash occurred on a curve, the slope of the road, and year. The variables shown here were variables that remained in the model. Complete case analysis was used (N = 173). McFadden’s R-square = 0.1008.

**Table 3:** Factors that predict male vs. female fatal, pedestrian, motor vehicle crashes in West Virginia, 2009–2019 ^a.^

Independent Variable	OR	95% CI	p
Pedestrian Positive for Drugs			0.023
No	1.00	Referent	
Yes	0.43	0.21, 0.89	
Season of Crash			0.135
Dec–Feb	1.00	Referent	
Mar–May	0.60	0.21, 1.68	
Jun–Aug	2.22	0.68, 7.20	
Sept–Nov	0.79	0.30, 2.07	
Rural Crash			0.015
No	1.00	Referent	
Yes	2.54	1.20, 5.38	
Roadway Curved			0.059
No	1.00	Referent	
Yes	3.13	0.96, 10.21	

Abbreviations: CI = confidence interval; OR = odds ratio; p = probability value; ^a^: A multivariable logistic regression model was run using backward selection with the *p*-value = 0.15 for an independent variable to remain in the model. Model initially included pedestrians’ ages, race, alcohol positivity, drug positivity, whether they were struck by an in-state driver, whether driver was previously cited, whether crash was a hit and run, vehicle type, season of crash, whether crash was on a weekday, time of crash, light conditions, weather conditions, road surface conditions, whether crash occurred on a highway, rurality, whether crash occurred at an intersection, speed limits, whether crash occurred on a curve, the slope of the road, and year. The variables shown here were variables that remained in the model. Complete case analysis was used (N = 173). McFadden’s R-square = 0.0648.

## Data Availability

The data used in this study are publicly available and can be accessed through the National Highway Traffic Safety Administration data portal at: https://www.nhtsa.gov/research-data/fatality-analysis-reporting-system-fars. accessed on 1 June 2021.

## Appendix A

**Table A1:** Characteristics of fatal pedestrian crashes which occurred in West Virginia: 2009–2019 stratified by rurality ^a.^

	Urban (N = 142)		Rural (N = 110)		Total (N = 252)	
Characteristic	Mean	SD	Mean	SD	Mean	SD
Survival Time (minutes)	1610	4887	418	2059	1074	3920
	N	%	N	%	N	%
Pedestrian Age (years)						
<20	9	6.3	8	7.3	17	6.8
20–29	26	18.3	18	16.4	44	17.5
30–39	26	18.3	24	21.8	50	19.8
40–49	27	19.0	25	22.7	52	20.6
50–59	25	17.6	14	12.7	39	15.5
≥60	29	20.4	21	19.1	50	19.8
Pedestrian Sex						
Male	91	64.1	87	79.1	178	70.6
Female	51	35.9	23	20.9	74	29.4
Pedestrian Race						
White	113	91.1	98	99.0	211	94.6
Non-white	11	8.9	1	1.0	12	5.4
Missing	18		11		29	
Pedestrian Positive for Alcohol						
No	83	64.3	67	66.3	150	65.2
Yes	46	35.7	34	33.7	80	34.8
Missing	13		9		22	
Pedestrian Positive for Drugs						
No	70	54.3	65	64.4	135	58.7
Yes	59	45.7	36	35.6	95	41.3
Missing	13		9		22	
Hit by an In-state Driver						
No	20	15.2	18	16.8	38	15.9
Yes	112	84.9	89	83.2	201	84.1
Missing	10		3		13	
Driver Previously Cited for Traffic Infractions						
No	73	56.6	58	55.8	131	56.2
Yes	56	43.4	46	44.2	102	43.8
Missing	13		6		19	
‘Hit and Run’ Crash						
No	113	79.6	93	84.6	206	81.8
Yes	29	20.4	17	15.5	46	18.2
Vehicle that Struck Pedestrian						
Car	44	33.6	31	29.3	75	31.7
Sport Utility Vehicle	32	24.4	21	19.8	53	22.4
Light Truck/Van	39	29.8	37	34.9	76	32.1
Other	16	12.2	17	16.0	33	13.9
Missing	11		4		15	
Season of Crash						
Dec–Feb	33	23.2	19	17.3	52	20.6
Mar–May	28	19.7	22	20.0	50	19.8
Jun–Aug	35	24.7	33	30.0	68	27.0
Sept–Nov	46	32.4	36	32.7	82	32.5
Day of Crash						
Mon–Fri	101	71.1	71	64.6	172	68.3
Sat–Sun	41	28.9	39	35.5	80	31.8
Time of Crash						
6:00 AM to 4:49 PM	29	20.7	29	26.4	58	23.2
5:00 PM to 5:59 AM	111	79.3	81	73.6	192	76.8
Missing	2		0		2	
Light Conditions						
Light	29	20.4	26	23.6	55	21.8
Low light	42	29.6	15	13.6	57	22.6
Dark	71	50.0	69	62.7	140	55.6
Inclement Weather						
No	117	82.4	89	80.9	206	81.8
Yes	25	17.6	21	19.1	46	18.3
Road Surface Conditions						
Dry	111	78.7	93	85.3	204	81.6
Wet/frozen/other	30	21.3	16	14.7	46	18.4
Missing	1		1		2	
Highway Crash						
No	33	23.2	33	30.0	66	26.2
Yes	109	76.8	77	70.0	186	73.8
Crash Occurred at Intersection						
No	117	84.8	93	92.1	210	87.9
Yes	21	15.2	8	7.9	29	12.1
Missing	4		9		13	
Roadway Speed Limit						
1–35 MPH	49	36.3	21	19.1	70	28.6
36–55 MPH	62	45.9	67	60.9	129	52.7
56 + MPH	24	17.8	22	20.0	46	18.8
Missing	7		0		7	
Roadway Curved						
No	121	85.2	91	82.7	212	84.1
Yes	21	14.8	19	17.3	40	15.9
Slope of Roadway						
Level	103	72.5	75	68.8	178	70.9
Grade/hill/sag	39	27.5	34	31.2	73	29.1
Missing	0		1		1	

Abbreviations: MPH = miles per hour; SD = standard deviation. a: Percentages may not add to 100% due to rounding. Two crashes were missing location.
